# Accuracy of the oscillometric method for the measurement of heart rate at rest and during mild exercise

**DOI:** 10.1097/HJH.0000000000002998

**Published:** 2021-08-30

**Authors:** Paolo Palatini, Ilaria Lazzaretto, Umberto Fontana, Claudio Fania

**Affiliations:** a Studium Patavinum, University of Padova; b Villa Maria Hospital, Padova, Italy

**Keywords:** device, exercise, heart rate, measurement, oscillometric

## Abstract

**Objective:**

Whether oscillometric devices provide accurate measurements of heart rate (HR) is not known. Our aim was to determine the accuracy of an oscillometric device for the measurement of HR during rest and exercise.

**Methods:**

The Microlife WatchBP O3, a monitor previously validated for blood pressure measurement, was tested in 36 individuals from the general population (mean age, 72.9 years; 18 men). HR was measured at rest and during stress testing on a cycle ergometer in order to increase HR by 15% or more. HR was computed from the oscillometric waves recorded during the deflation phase of the blood pressure measurement and was compared with HR measured by pulse palpation by two observers.

**Results:**

At rest, the mean device–observer HR difference was 0.9 ± 2.1 bpm. During exercise, the average HR increase was 20.3% and the mean device–observer difference was 0.6 ± 2.6 bpm. The device–observer HR differences were all included within ±6 bpm both at rest and during exercise. Kappa statistics showed a very good agreement between device and observers both at rest (kappa scores, 0.82–0.88) and during exercise (kappa scores, 0.81 and 0.86). The device–observer HR differences were unrelated to the level of HR or to pulse pressure.

**Conclusion:**

The present study shows that the oscillometric technique is able to provide reliable HR measurements during rest and mild exercise. Whether evaluation of HR performance should be included during validation testing of automatic monitors should be established by regulatory bodies.

## INTRODUCTION

In the three last decades, a large number of automatic devices for blood pressure (BP) measurement mainly based on the oscillometric method have been introduced into the market [1]. The widespread use of BP measurement devices has called for strict criteria to test their accuracy according to international protocols [2,3].

In addition to yielding BP readings, oscillometric devices also provide heart rate (HR) measurements using the oscillometric signal. HR is increasingly being recognized as an important risk factor for cardiovascular disease [4,5]. This has been shown by a large number of epidemiologic studies and clinical trials, which have often used HR measured with oscillometric devices to demonstrate its prognostic value [6–9].

Despite the widespread use of HR measured with oscillometry both in clinical practice and research studies, no criteria for the validation of HR were included in the device validation protocols. This concern applies especially to devices designed for measuring BP and HR in ambulatory conditions during which HR can vary over a wider range and incorrect readings because of artefacts are more likely to occur. This issue has been overlooked by the literature and no study has attempted to test the reliability of HR measured with oscillometric devices using standardized criteria. Also in the recent International Organization for Standardization (ISO) protocol 81060–2:2018, validation of HR measurement was not considered [3].

To fill this gap in knowledge, the present study compared the HR readings provided by the monitor WatchBP O3, an oscillometric device designed for 24-h ambulatory BP recording [10], with HR measurements obtained by pulse palpation by two observers in resting conditions and during mild exercise.

## PARTICIPANTS AND METHODS

This investigation was conducted in the participants of a study on the performance of the WatchBP O3 for BP measurement [10]. The results showed that the monitor satisfied the ISO 81060–2:2018 standard requirements for a general population both at rest and during exercise.

### Participants

Thirty-six participants older than 12 years of age (range 30–91) were recruited from among the outpatients or the staff of the Villa Maria Hospital, Padua, Italy. Six of the participants were healthy normotensive volunteers. Among the 30 outpatients, 66.7% had hypertension on antihypertensive treatment, 26.7% had chronic kidney disease, 20% were diabetic, 16.7% had coronary artery disease, and 13.3% were obese (BMI ≥30 kg/m^2^). Participants with atrial fibrillation or other rhythm irregularities were not included. The sample size was established based on the test requirement of the ISO81060–2:2018 standard where the minimum sample size required for a device intended for use in ambulatory conditions is of 35 participants [3]. Mean resting HR at enrolment was 74.7 ± 9.2 bpm and mean BP was 134.3 ± 17.3/81.4 ± 13.6 mmHg. Fifty percent were men. The study was approved by the Institutional Review Board of the Villa Maria Hospital and was performed according to the Declaration of Helsinki. A written informed consent was given by all the participants.

### Device

The Microlife WatchBP O3 model is an oscillometric fully automatic device for ambulatory BP measurement at the upper arm. Its characteristics have been described previously [10]. The manufacturer supplied two test devices and confirmed that they had been selected from a normal production line. In addition to providing SBP and DBP readings, the device also measures HR, which is displayed on a liquid crystal digital display together with BP values.

### Heart rate measurement

**FIGURE 1:**
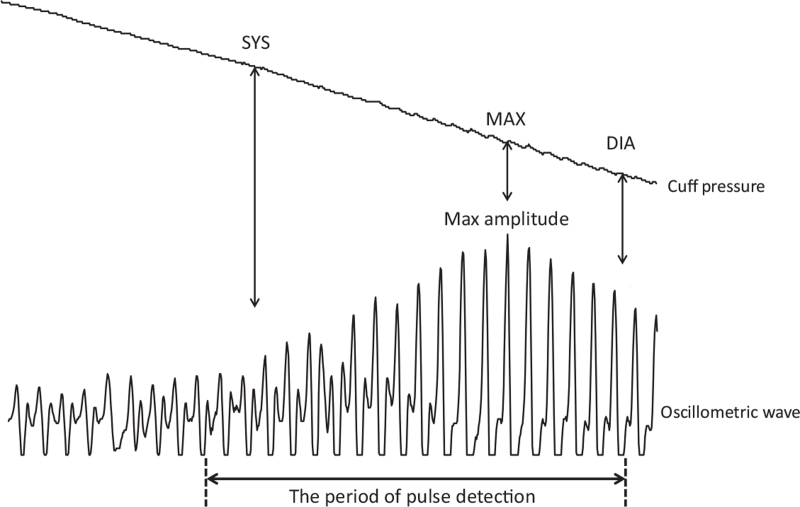
Algorithm used by the device for the measurement of heart rate. The software calculates the average interval of all pulse intervals during a measurement cycle. The period of pulse detection starts a few pulses before the systolic point and ends when the pulse amplitude goes below a certain value, typically a few pulses after diastolic point. If a pulse interval is more than or less than *x*% of the average, it is deleted by the software and the average of all remaining pulse intervals is recalculated. SYS, systolic point; DIA, diastolic point; MAX, maximal wave amplitude corresponding to mean blood pressure.

The algorithm calculates the average interval of all pulse intervals during a measurement cycle (Fig. 1). Pulse detection during the deflation phase starts a few pulses before the systolic point and ends when the pulse amplitude goes below a certain value, typically a few pulses after the diastolic point. Then each pulse interval is compared with the average of all pulse intervals. If a pulse interval is more than or less than *x*% of the average, it is deleted and the average of all remaining pulse intervals is calculated. The number of pulse intervals taken into account may vary in relation to the individual HR and pulse pressure levels. Typically, with a deflation rate of 4 mmHg/s, 10–20 pulse intervals are averaged during a measurement cycle. If the number of pulse intervals is lower than 7, the HR measurement is discarded.

### Procedures

The clinical study was performed by two trained observers. HR was measured by pulse palpation over 30 s. For both observers, the coefficient of variation from 30 duplicate measurements was 2% or less for HR measured at rest and 4.3% or less for HR measured during steady-state mild exercise. Validation was carried out performing sequential same-arm measurements following the recommendations of the ISO81060–2:2018 standard for BP measurement [3]. Four sequential HR measurements were taken by observers 1 and 2 (OHR1, OHR3, OHR5, OHR7), and three HR readings were taken by the supervisor with the test device (DHR2, DHR4, DHR6). Each of the test device measurements was compared against the average of the previous and next reference OHR reading. (e.g. DHR2 versus the average of OHR1–OHR3, DHR4 versus average of OHR3–OHR5, DHR6 versus average of OHR5–OHR7). Each of the reference HR measurements was the average of the simultaneous readings taken by the two observers. Differences were calculated by subtracting the reference HR measurement from the test device measurement. A different procedure was performed during exercise because of the changing HR levels during the two exercise steps. Soon after HR increased by 10% or more, two HR observers’ measurements were made, one before and one after the device measurement. The same procedure was repeated after HR increased by 15% or more.

### Statistical analysis

To compare HR measured by the device versus HR measured by the observers, the Bland–Altman limits of agreement were generated. The device–observer HR differences were expressed as mean ±SD, and the percentage of differences within ±5 bpm was calculated. The data were also evaluated using kappa statistics, which measure agreement occurring in excess of that expected by chance. Kappa was calculated according to the Cohen's method [11] and the standard error and 95% confidence interval (95% CI) were calculated according to Fleiss *et al.*[12]. According to Altman [13], the strength of agreement can be defined as poor if kappa is less than 0.20, fair if kappa is 0.21–0.40, moderate if kappa is 0.41–0.60, good if kappa is 0.61–0.80, and very good if kappa is 0.81–1.00.

Correlations between the device–observer differences and pulse pressure or the HR level were made using the Pearson test. Analyses were performed using Systat version 12 (SPSS Inc., Evanston, Illinois, USA). MedCalc version 19 (MedCalc Software, Ostend, Belgium) was used for kappa statistics and to generate the Bland–Altman plots.

## RESULTS

### Data at rest

**FIGURE 2:**
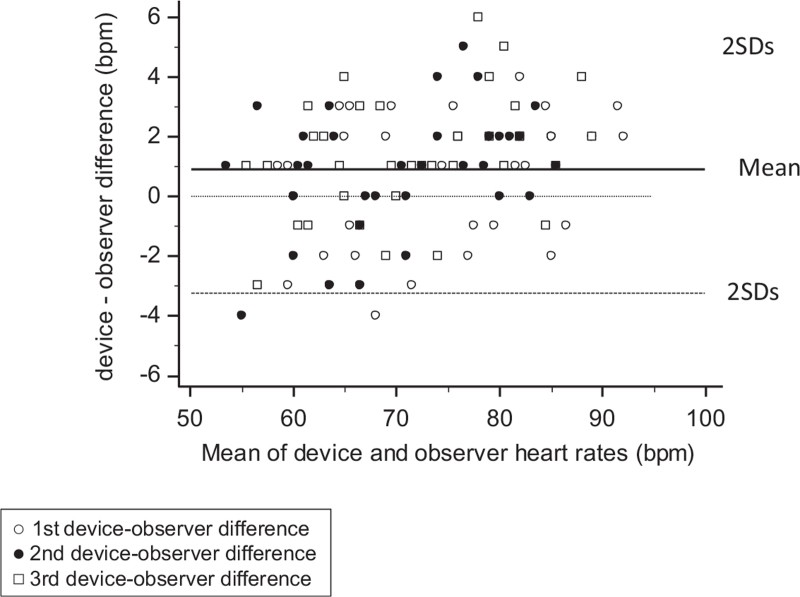
Bland--Altman scatter plots of the heart rate differences between the device and the observers (*y*-axis) against the average of the test device and observer heart rate values (*x*-axis) in the participants studied at rest. Three sets of comparisons are displayed.

Mean observers’ HR in resting conditions (mean of 288 readings) was 72.1 ± 9.3 bpm. The difference between the two observers was 0.3 ± 2.0 bpm. The mean device–observer difference in the 108 separate HR data pairs was 0.9 ± 2.1 bpm. Bland–Altman plot of the device–observer HR differences is shown in Fig. 2. All but one differences were within ±5 bpm. According to kappa statistics, all comparisons at rest gave a kappa index greater than 0.80 with low standard errors (Table 1).

**TABLE 1:** Cohen's kappa statistics for agreement between heart rate measured by the device and heart rate measured by the observers at rest and during exercise

Device-observer	Kappa index	Standard error	95% CI
RestFirst comparison	0.82	0.021	0.78–0.86
RestSecond comparison	0.87	0.015	0.84–0.90
RestThird comparison	0.88	0.018	0.84–0.91
ExerciseHR greater than 10%	0.86	0.024	0.82–0.91
ExerciseHR greater than 15%	0.81	0.031	0.75–0.87

HR indicates increase in heart rate from rest to exercise. CI, confidence interval.

### Data during exercise

**FIGURE 3:**
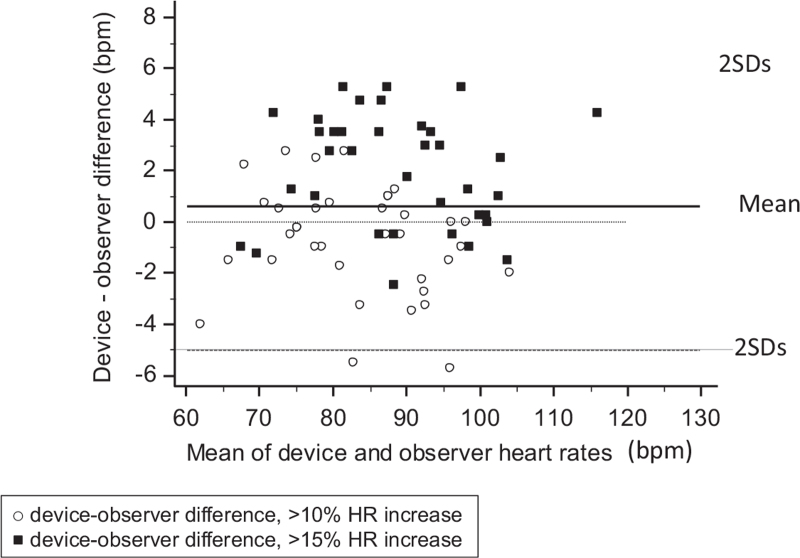
Bland--Altman scatter plots of the heart rate differences between the device and the observers (*y*-axis) against the average of the test device and observer heart rate values (*x*-axis) in the participants studied during bicycle ergometry. Two sets of comparisons made during the two exercise stages are displayed.

The minimum HR increase during exercise stress testing (15%) was obtained in all participants. During exercise, HR measured by the observers was 83.7 ± 10.5 soon after the greater than 10% HR increase, and was 88.0 ± 11.1 bpm after more than 15% HR increase. The difference between the two observers was 0.3 ± 1.7 bpm. Bland–Altman plot of the device–observer HR differences is shown in Fig. 3. The mean exercise device–observer difference in 72 separate HR data pairs was 0.6 ± 2.6 bpm. Sixty-seven data points (93.1%) fell within ±5 bpm and all data points within ±6 bpm. The kappa index was 0.86 for exercise HR greater than 10% and was 0.81 for exercise HR greater than 15% (Table 1).

### Correlations

At rest, the device–observer HR difference was unrelated to HR measured by the observers (*r* = 0.15, *P* = 0.12) and to pulse pressure (*r* = 0.01, *P* = 0.90). A lack of correlation of the device–observer HR difference with HR level and pulse pressure was also found during exercise (*r* = −011, *P* = 0.37 and *r* = −0.02, *P* = 0.84, respectively).

## DISCUSSION

The present results show that the oscillometric technique is able to provide accurate HR measurements both at rest and during exercise. The HR differences between the device and the mean of the two observers were less than 1 bpm in both conditions and the standard deviations of the differences were less than 3 bpm. In addition, kappa statistics showed very good agreement between HR measured by the device and the observers both at rest and during exercise.

Automatic monitors are designed for measuring BP at the upper arm or the wrist but they can also provide simultaneous HR data. However, all protocols for the validation of these devices provide strict criteria for testing the reliability of BP disregarding the measurement of HR [2,3]. Thus, it is not known if the oscillometric method is actually able to provide accurate HR data as no monitor used in previous studies passed a validation test according to standardized criteria.

HR is increasingly being considered as an important predictor of risk in hypertensive patients [4,5,14]. In the latest ESH Consensus document on the management of the hypertensive patient with elevated HR, it is recommended that HR be measured by pulse palpation over 30 s or with electrocardiography [15]. The issue of HR measurement by automatic monitors was not raised.

In a previous study, Palma *et al.*[16] tested the reliability of the Omron HEM 742, an oscillometric electronic arm device, in a group of women. The authors found good agreement between HR measured with the oscillometric device and HR obtained with the Polar RS800 CX, a device whose reliability for measuring HR had been questioned by several authors [17,18]. In addition, no information on the methods used to compare the readings provided by the two devices was provided. In other studies, some authors found a moderate positive correlation between HR measured with the oscillomteric method and HR simultaneously measured with the electrocardiogram but these investigations were made in individuals with atrial fibrillation [19,20].

Despite the lack of evidence to support the accuracy of oscillometric devices, HR measured with oscillometric monitors has been frequently used to investigate its association with adverse outcomes either in observational cohort studies [6] or in clinical trials [7]. Prognostic studies used oscillometric HR not only measured at rest but also during 24-h ambulatory monitoring [8,9] during which HR is subject to greater variability.

### Methodological issues

Due to the lack of official criteria to establish the validity of oscillometrically measured HR, in the present study, we used the procedures and the criteria of the recent ISO protocol 81060–2:2018 designed to test the accuracy of BP measured with automatic monitors [3]. According to this protocol, the mean device–observer BP difference should be 5 mmHg or less and its SD 8 mmHg or less for both resting and exercise BPs. In the present study, both criteria were comfortably fulfilled by HR in either condition and the device–observer HR differences were all included within ±6 bpm. In addition, Cohen's kappa statistics showed that kappa index was at least 0.82 for all between-method comparisons at rest, a score that can be defined as ‘very good’ according to Altman's classification [13]. A very good agreement was found also during mild exercise. although the kappa score was a little smaller for a HR 15% higher or more than resting values.

The number of oscillometric waves taken into account by the device during each measurement depends on the level of HR and pulse pressure and usually varies from 10 to 20. As a consequence, in individuals with bradycardia and low pulse pressure, a smaller number of waves will be included in the oscillometric waveform envelope. For this reason, we wanted to test whether the device performance might be influenced by these two clinical variables. However, no correlation was found between the device–observer HR differences and the level of HR or pulse pressure.

### Limitations

Despite the overall positive findings of this study, some limitations should be mentioned. We used arbitrary criteria to evaluate the accuracy of HR measured with the oscillometric device as no established standards are currently available. In addition, the lack of a gold standard for assessing the reliability of the oscillometric technique does not allow firm conclusions to be made. However, we employed a rigorous methodology to compare oscillometric HR with HR measured by the observers [3] and the results were quite reassuring. Another limitation may be the relatively small number of participants included in the study. However, this is the sample size required by the ISO 81060–2:2018 protocol for the validation of ambulatory BP monitors [3]. Finally, although the device performance was also tested during exercise, the maximum increase in HR was only ∼20% of resting values. However, our goal was not to validate the device for its use during stress testing but to simulate the hemodynamic changes that usually occur during ambulatory measurements.

The present study shows for the first time that the oscillometric method is able to provide reliable HR measurements during rest and mild exercise. However, the results obtained with the Microlife WatchBP O3 monitor do not necessarily apply to other oscillometric devices as the reliability of the data depends on the software performance as well as on its operational usage. Whether evaluation of HR performance should be included during validation testing of automatic monitors for BP measurement should be established by regulatory bodies.

## ACKNOWLEDGEMENTS

This work was funded by a grant from Microlife AG, Espenstrasse 139, CH 9443, Widnau, Switzerland.

### Conflicts of interest

There are no conflicts of interest.
